# Habitat and seasonal drivers of leukocyte profiles within and across Neotropical bat species

**DOI:** 10.1098/rsbl.2025.0447

**Published:** 2025-10-29

**Authors:** Daniel J. Becker, Kristin E. Dyer, Lauren R. Lock, M. Brock Fenton, Nancy B. Simmons

**Affiliations:** ^1^ School of Biological Sciences, University of Oklahoma, Norman, OK, USA; ^2^ Department of Biology, Western University, London, Canada; ^3^ Department of Mammalogy, American Museum of Natural History, New York, NY, USA

**Keywords:** ecommunology, haematology, phylogenetic comparative methods, Chiroptera, land conversion

## Abstract

Land conversion is a widespread form of environmental change that can alter infection dynamics in wildlife by modifying host immune defence. Such effects may be compounded by seasonal variation in resources and reproduction and differ among members of a host community, yet the combined effects of habitat, season and species identity on wildlife immunity remain poorly understood. We tested within- and across-species effects of land conversion and seasonality on immunity in Neotropical bats by quantifying haematological markers of physiological stress and inflammation. We sampled seven species across a large forest preserve and smaller nearby forest fragment in northern Belize during the dry and wet seasons. Using phylogenetic generalized linear mixed models, we tested overall effects of habitat and season and quantified per-species impacts. Total leukocyte counts and neutrophil-to-lymphocyte ratios showed no overall habitat or seasonal effects but displayed strong species-specific responses to these predictors. In contrast, the systemic inflammation response index was higher across species in the dry season and in the smaller fragment, suggesting poor health in unfavourable conditions. Species-specific effects did not align with diet guilds, indicating potential roles for finer-scale ecological traits. Our results highlight the complex, species-dependent effects of environmental change on wildlife immunity.

## 1. Introduction

Land conversion is one of the dominant and accelerating forms of contemporary environmental change [1], with cascading effects on wildlife population and community dynamics [2,3]. Such changes have also have driven increased infectious disease risks within wildlife hosts and, through zoonotic spillover, to humans [4–6]. One of the primary proposed mechanisms by which land conversion alters infection dynamics is through impacts on wild host immunity [7,8]. Decreased amount or quality of space and food resources in degraded or fragmented habitats can increase physiological stress, such as by expending more energy during foraging or increasing frequency of negative intra- or interspecific interactions (e.g. crowding or edge effects) [9,10]. Prolonged physiological stress (i.e. allostatic overload) can in turn affect host immune responses [11,12], including increased susceptibility to new infections and the relapse of chronic infections [13,14].

The immunological consequences of land conversion could be further compounded by seasonality [8]. Seasonal reproductive activity can favour allocation of energetic resources away from immune defences in mammals, particularly when females are pregnant or lactating [15,16]. Similarly, periods with less-favoured climatic conditions or reduced food availability, such as winters in temperate regions or the dry season in tropical regions, can also impose energetic costs and limit energy allocation to immunity [17–19]. As such, habitat degradation or fragmentation could have the most pronounced effects on host defences during birth pulses or these unfavourable periods, given further increases to energy required for homeostasis and amplifying trade-offs between immunity and other physiological functions [20,21]. For example, such interactive effects were hypothesized to explain why pulses of Hendra virus shedding from Australian flying foxes occur primarily in the winter months and for colonies that recently experienced food shortages and were displaced into agricultural and urban habitats [22]. However, the immunological underpinnings of such relationships remain poorly understood, partly because their study requires field sampling designs that jointly capture the effects of both habitat and seasonality [23]. More even host sampling across habitats and among seasons could test hypotheses about interactive effects and reveal where and when hosts are susceptible [24].

Finally, although first principles surrounding energy expenditure and allocation suggest overall deleterious impacts of land conversion and unfavourable seasons on wildlife immunity, this outcome may not be homogeneous across host species. As emphasized through trait-based approaches to studies of global change [25], species do not respond equally to increasing anthropogenic pressures, even if mean effects are often negative [26–28]. For example, the demography and distribution of some generalists are less affected by habitat fragmentation than specialists [29,30], suggesting such species could show less immunological change in converted habitats. Many generalists can capitalize on new resources provided in these altered landscapes, potentially allowing greater allocation of energy into immunity [31,32]. For example, white ibis occupying urban habitats feed more consistently on anthropogenic resources and, in turn, have lower glucocorticoid levels and stronger innate immunity [33,34]. From a similar perspective, species that rely on more ephemeral resources, such as frugivores or nectarivores, may be especially physiologically vulnerable to seasonal shifts in food resources [35], while species with generalist foraging behaviour could instead show less seasonal variation in immunity [36]. Given such complexities, there is a need for spatially and temporally explicit field studies that assess immune impacts within not only a single host species but also across the broader community.

We here provide an initial test of the within- and across-species effects of land conversion and seasonality on immunity in the context of Neotropical bats. Bats (order Chiroptera) have been increasingly studied as model systems for comparative immunology [37], given multiple immune adaptations that differ from other mammals and substantial inter-specific variation in defence [38,39]. Bats display species-specific responses to land conversion [40,41], with especially pronounced effects in the Neotropics [42]. The Neotropics contain some of the most extreme concentrations of bat diversity globally [43], driven in part by diversification through dietary shifts [44,45]. Neotropical bats encompass a wide range of foraging strategies, including not only frugivory, nectarivory and insectivory but also carnivory, piscivory and haematophagy [46,47]. Land conversion for cropland and pasture is also widespread and accelerating in the Neotropics [48,49], with the responses of bat demography and occupancy dependent on foraging ecology [50,51]. Diet also plays a role in immune differences among Neotropical bat species, their physiological responses to resource availability and whether they display unimodal or bimodal reproductive phenologies [52–56]. As such, Neotropical bats are an ideal system for testing species-specific responses of host immunity to both land conversion and seasonality.

We focused our analyses on a long-term research programme in northern Belize (Orange Walk District), where increasing habitat fragmentation driven by agricultural change has led to pronounced shifts in bat communities [57–59]. Surveys at the Lamanai Archaeological Reserve (LAR) and Ka’Kabish (KK), which comprise a large preserve and a small, isolated fragment, respectively, indicate the KK bat community is a nested subset of that found in the LAR [59]. Our prior work here has shown site-level differences in contaminant dynamics (in part driven by agricultural change) that impact bat immunity [60] as well as select species-specific responses of bat immunity to ongoing habitat fragmentation in the region for bats occurring in the LAR [61]. Other efforts have focused on immunological characterization of those species that occur in both sites, such as *Desmodus rotundus*, *Artibeus jamaicensis* and *Pteronotus mesoamericanus* [62–65]. By systematically sampling common bat species in both the LAR and KK and across two seasons, we test the hypothesis that bat immune systems, as revealed by haematology, would show signatures of physiological stress and inflammation that are most pronounced in the smaller habitat and unfavourable season. At the same time, we predicted that bat species would also vary in their immunological response to both habitat and season in ways that align with foraging ecology, such as by those that can capitalize on year-round prey in agricultural habitats (e.g. insectivorous and haematophagous bats) being less physiologically affected by agriculture and seasonality, while frugivorous and nectarivorous species dependent on forest plants could be especially immune impaired in unfavourable environments owing to reduced food availability.

## 2. Methods

### (a) Bat sampling

During two-week periods in November 2021 and April–May 2022, we sampled bats in both the LAR and KK as part of broader studies of intra- and interspecific variation in immunity and pathogen diversity [60,61,66]. These periods coincided with the late wet season (November) and the height of the dry season (April–May) [67], with climatic differences being driven mostly by temperature rather than by precipitation (electronic supplementary material, figure S1). Bats were captured using mist nets and harp traps along flight paths, held in individual bags and identified to species based on morphology [59,68]. We also recorded sex, reproductive status and age [69]. We collected blood using heparinized capillary tubes after lancing the propatagial vein with sterile needles (26−30 G); all blood volumes were less than 1% body mass. Thin blood smears were prepared on glass slides, air dried in the field and then stained with Wright–Giemsa (Quick III Set, Astral Diagnostics Inc.) at the University of Oklahoma. All bats for this study were released following sampling.

### (b) Haematological analyses

We used blood smears to quantify total and differential white blood cell (WBC) counts. Such measures require only 1−2 μl blood, are cost effective and do not depend on species-specific reagents, making them amenable to fieldwork conditions and studies of small vertebrates such as bats [70–72]. However, we note that such counts provide only simplified and coarse insights into immunity and do not quantify functional activity or specific cell subsets [73,74]. Using a light microscope, we estimated total WBC counts as the mean number of leukocytes from 10 fields of view (400×) [54]. We next performed differential counts (1000×, oil immersion) to quantify the relative abundance of neutrophils, lymphocytes, monocytes, eosinophils and basophils from 100 WBCs [75]. We used our differential WBC counts to derive haematological indices of physiological stress and inflammation, including the neutrophil-to-lymphocyte ratio (NLR) and the systemic inflammation response index (SIRI, the ratio of the neutrophil and monocyte quotient to lymphocytes) [76–78]. One observer (K.E.D.) analysed all blood smears.

### (c) Statistical analyses

From a broader sample of 294 bats (*n* = 21 species) with haematology data, we focused analyses on species captured in both the LAR and KK and with a sample size greater than or equal to nine (*n* = 232 individuals spanning seven species). These seven species included *D. rotundus* (*n* = 77), *A. jamaicensis* (*n* = 48), *Sturnira parvidens* (*n* = 29), *P. mesoamericanus* (*n* = 25), *Glossophaga mutica* (*n* = 23), *Carollia sowelli* (*n* = 21) and *Saccopteryx bilineata* (*n* = 9), representing one haematophagous species (*D. rotundus*), three frugivorous species (*A. jamaicensis*, *St. parvidens* and *C. sowelli*), one nectarivorous species (*G. mutica*) and two insectivorous species (*P. mesoamericanus* and *Sa. bilineata*). All species belong to the family Phyllostomidae with the exceptions of *P. mesoamericanus* (Mormoopidae) and *Sa. bilineata* (Emballonuridae; electronic supplementary material, figure S2). Per-species sample sizes varied among seasons and sites (electronic supplementary material, figure S3), and species varied in the seasonal distribution of male and female reproductive activity (electronic supplementary material, figure S4). Within this dataset, our haematology responses were not correlated (*ρ* = −0.14–0.54, x̄ = 0.15).

We used phylogenetic generalized linear mixed models (PGLMMs) fit using the *brms* package in R to test effects of site and season on haematology, while controlling for evolutionary history [79,80]. For each of our haematological outcomes (total WBC count, NLR and SIRI), we included the above fixed effects alongside sex, reproductive status and age. We also included the time between capture and blood collection (i.e. holding time) to account for impacts of this acute stressor [76,81]. Because a small number of bats lacked data on reproductive status (*n* = 5), age (*n* = 5) or holding time (*n* = 9), we used the *missRanger* package to impute these missing values using chained random forests (*n* = 1000 trees) [82,83]. We assessed collinearity by fitting a linear model with the same fixed effects, finding consistently low variance inflation factors (1.04−1.48). To account for species-specific responses, each model included a random intercept of species and random slopes of site and season [84]. We also included a phylogenetic random intercept using a variance–covariance matrix derived from the bat phylogeny using the *ape* package [85,86]. We did not estimate the correlation between random slopes and intercepts nor included phylogenetic random slopes to avoid overparameterization and to improve PGLMM convergence [87]. All models used Gaussian errors with log_10_-transformed response; owing to zero values for the total WBC count and SIRI, we added half the minimum non-zero value prior to transformation [88]. We used leave-one-out information criterion (LOOIC) to compare our base model with a model that also included the interaction between site and season [89]. We ran our models using four chains for 12 500 iterations and a burn-in of 50%, thinned every 25 steps. We verified model convergence by inspecting trace plots and $\hat{\text{R}}$ values. We then summarized the posterior mean and corresponding 95% credible interval for each PGLMM coefficient, including species-specific slopes for both site and season, using the *ggdist* and *tidybayes* packages [90]. Finally, we also assessed both marginal and conditional *R^2^* with the *performance* package [91,92].

## 3. Results

**Figure 1.**
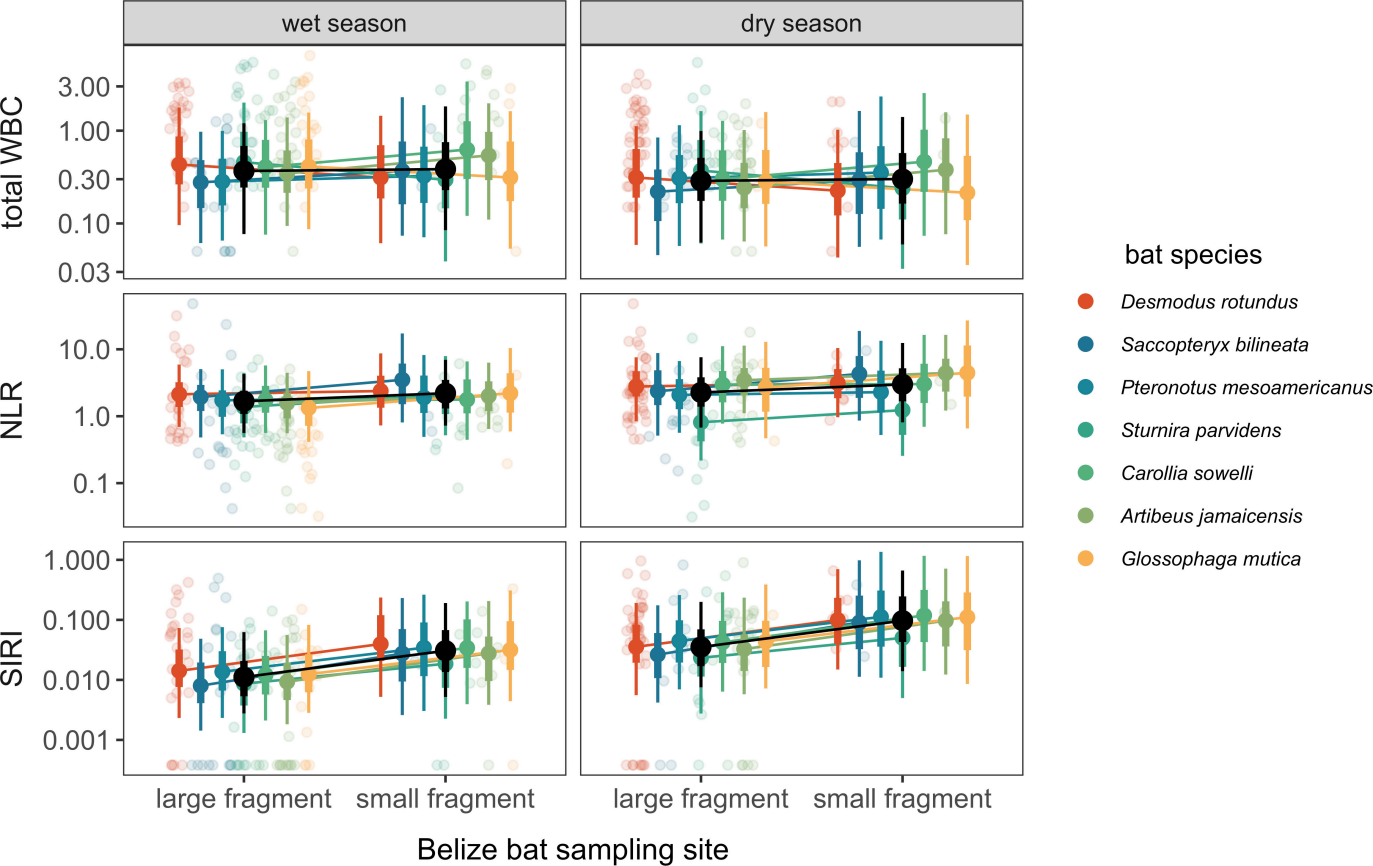
Posterior means and credible intervals (highest density intervals, 66% and 95%) are shown for the additive effects of habitat and season (black) for all three haematology PGLMMs. Species-specific responses derived from each model’s random effects are also shown, with raw data overlaid and jittered to reduce overlap. Haematology values are displayed on a log_10_ scale.

Across all three haematology outcomes in our sample subset of the Belize bat community, PGLMMs with additive effects of site and season were always supported by LOOIC over models with interactive effects (electronic supplementary material, table S1). The fixed effects explained 10−22% of the variation in our haematology variables, while the species-level random effects explained an additional 5−15% of the variance. For total WBC counts and the NLR, we found no overall effect of site or season (table 1, figure 1). However, males had higher total WBC counts, and reproductively active bats had lower total WBC counts and elevated NLRs. Our models found no baseline difference in the total WBC count nor species-specific responses to seasonality (i.e. low variation in the species-level random intercepts and in the random slopes of season), whereas we did find species-specific responses to site (i.e. non-zero random slopes of habitat). We also detected baseline differences in the NLR and species-specific responses to season (i.e. non-zero random intercepts of species and random slopes of season) but no species-specific responses to site (i.e. low variation in the random slopes of site). In contrast, the SIRI did show overall effects of both site and season, with this measure of inflammation being elevated in KK and the dry season (table 1, figure 1). Bats also displayed species-level differences in baseline SIRI measures (i.e. a non-zero random intercept of species), whereas the SIRI did not show species-level variation by site or season (i.e. low variation in random slopes). None of our models found strong age or residual phylogenetic effects, and only the NLR showed a positive association with holding time.

**Table 1.** Posterior means and 95% credible intervals (highest density intervals) of fixed and random effects from the top PGLMMs for all three haematological response variables in Belize bats. Parameters for which 95% credible intervals do not cross zero are displayed in bold.

effects	parameter	total WBC	NLR	SIRI
fixed (β)	intercept	−0.28 (−0.75, 0.37)	**−0.47 (−0.96, −0.03**)	**−2.36 (−3.03, −1.56**)
	KK	0.02 (−0.28, 0.34)	0.12 (−0.14, 0.42)	**0.44 (0.03, 0.86**)
	dry season	−0.11 (−0.31, 0.08)	0.13 (−0.21, 0.45)	**0.50 (0.15, 0.83**)
	males	**0.20 (0.09, 0.32**)	−0.01 (−0.14, 0.13)	−0.11 (−0.37, 0.13)
	adult	−0.04 (−0.20, 0.16)	0.16 (−0.02, 0.37)	0.03 (−0.35, 0.40)
	reproductive	**−0.16 (−0.31, −0.01**)	**0.35 (0.20, 0.52**)	0.27 (−0.06, 0.58)
	holding time	0.06 (−0.20, 0.28)	**0.28 (0.04, 0.60**)	0.16 (−0.37, 0.67)
random (s.d.)	1|species	0.209 (0, 0.519)	**0.176 (0.001, 0.429**)	**0.248 (0.001, 0.637**)
	site|species	**0.301 (0.004, 0.687**)	0.217 (0, 0.559)	0.254 (0, 0.698)
	season|species	0.151 (0, 0.377)	**0.324 (0.005, 0.721**)	0.216 (0, 0.55)
	1|phylogeny	0.048 (0, 0.109)	0.035 (0, 0.083)	0.052 (0, 0.119)

**Figure 2.**
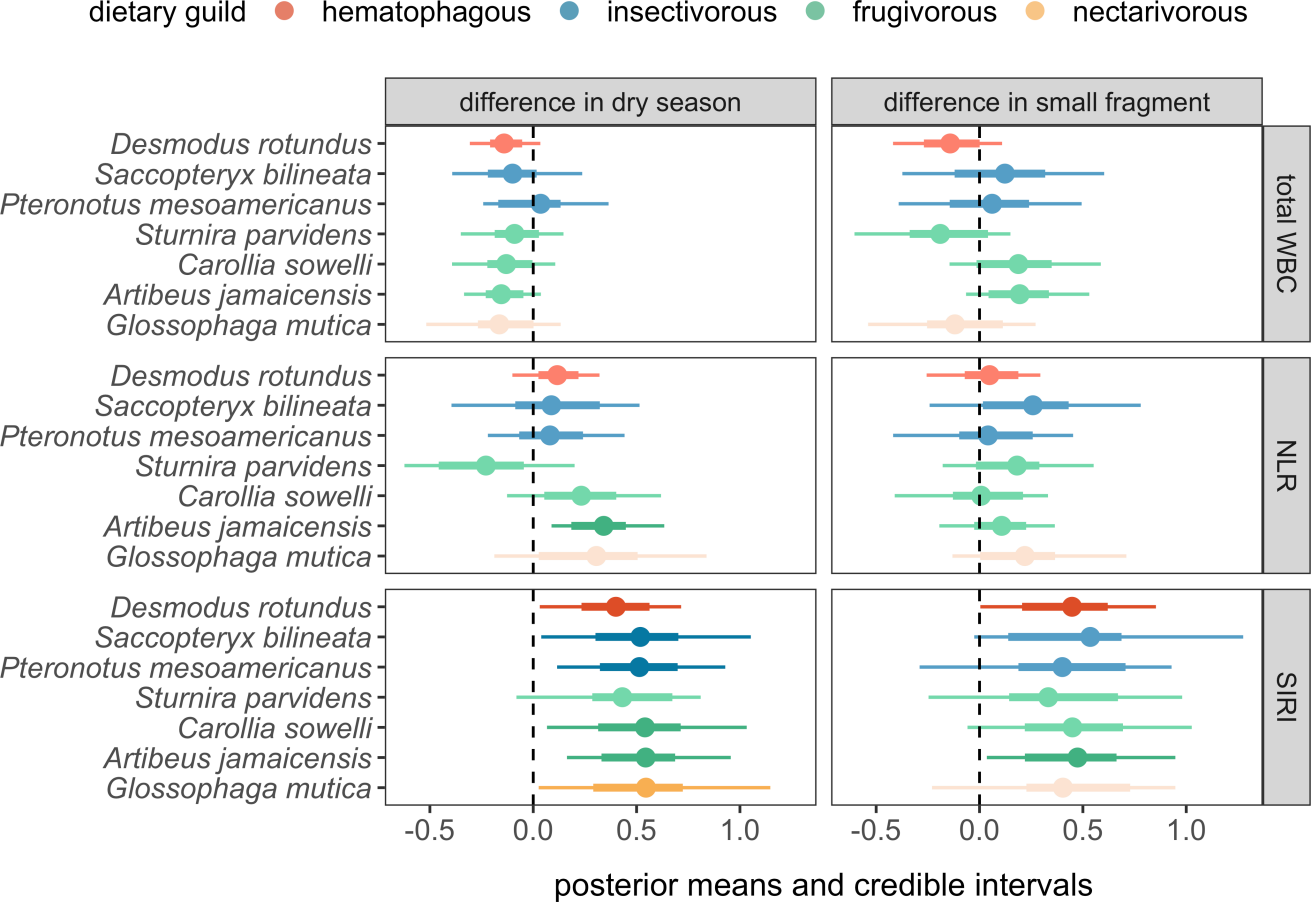
Posterior means and credible intervals (highest density intervals, 66 and 95%) are shown for the species-specific habitat and season coefficients derived from each haematology PGLMM. Effects are coloured by dietary guild, with darker shading indicating those effects in which the 95% credible intervals do not overlap with zero (dashed vertical line). Estimates are presented after statistically adjusting for sex, reproductive status, age and log_10_-holding time.

Although bats showed species-specific haematological responses to season (i.e. NLR) and site (i.e. total WBC counts), these effects did not map onto dietary guild (figure 2). Most species had fewer WBCs in the dry season, with the exception of *P. mesoamericanus*; however, 95% credible intervals for all dry season effects crossed zero. In contrast, most species had elevated NLRs in the dry season, with this effect strongest for *A. jamaicensis*; only *St. parvidens* had lower NLRs during this period. Given the negligible random slopes of season for SIRI and strong overall effect, all bats had elevated inflammation in the dry season, although effects were strongest for *P. mesoamericanus*, *C. sowelli* and *A. jamaicensis*. Occupying KK had variable species-specific effects for the total WBC count, with both insectivorous species, *C. sowelli*, and *A. jamaicensis* having more leukocytes, while *D. rotundus*, *St. parvidens* and *G. mutica* had fewer leukocytes (although 95% credible intervals for these KK effects crossed zero). Most bats had weakly higher NLRs in KK, with effects strongest for *Sa. bilineata* and *G. mutica* (all 95% credible intervals again crossed zero). Given the overall effect of the site on the SIRI, all species had elevated inflammation in KK, but this contrast was strongest for *D. rotundus* and *A. jamaicensis* (whereas the 95% credible intervals crossed zero for all other species).

## 4. Discussion

Although land conversion is hypothesized to affect infectious disease risks through changes in wild host allostatic load and, in turn, immune defence [7,8,10,21], how these effects are modified by seasonal variation in wildlife energetics as well as by host ecology and evolution remains poorly understood. Here, we leveraged a long-term study system on diverse Neotropical bats in Belize to partition the within- and across-species effects of habitat fragmentation and seasonality on cellular immunity. While some haematological indices did display consistent effects of site and season (e.g. the SIRI), other measures showed stronger species-specific responses to these drivers. However, these species-specific effects did not align clearly with dietary guild, indicating roles for finer-scale traits describing foraging ecology or other characteristics such as reproductive phenology or roosting ecology. Together, our findings demonstrate the complex effects of land conversion on wildlife’s immune defence and highlight important next steps to improve our understanding of how environmental change ultimately affects infection risks.

Our work provides general support for plausible immune impairment in fragmented habitats and the dry season. The SIRI was elevated in both contexts across the bat community (i.e. 95% credible intervals for fixed effects did not include zero), although effect sizes were relatively small. The SIRI is a biomarker of inflammation validated in human cancer studies, involving neutrophilia, lymphopenia and monocytosis as well as increased levels of some inflammatory cytokines [77]. A relative increase in neutrophils and decrease in lymphocytes in blood (i.e. increased NLR) can indicate prolonged physiological stress, whereas increases in the relative abundance of monocytes can indicate acute infection [76]. We found no overall increase in the NLR in the smaller fragment or dry season, although work in captive vertebrates suggests that monocytosis can also result from stressors including heat, crowding and pregnancy [93–96]. While we cannot discount that elevated infection risks, especially with known bacterial and protozoan pathogens common in this study system [60,62], could cause observed habitat and seasonal differences in the SIRI, such drivers could also result from feedback between poor condition and infection [97]. More broadly, our findings are consistent with the hypothesis that energetic trade-offs and allostatic load can be exacerbated by land conversion and unfavourable periods, resulting in inflammation and immune dysregulation [7,8,21]. Our overall seasonal results may also partly reflect reproduction, as reproductive activity was associated with lower total WBC count, higher NLR and weakly higher SIRI across bat species; further, reproductive activity across males and females was more often observed in the dry season (69%) compared with the wet season (41%; electronic supplementary material, figure S4). Such findings align with trade-offs between reproduction and immunity in wildlife, including but not limited to bats [15,16,71,98,99], although direct effects of climate and indirect effects of resource limitation during the dry season cannot be discounted.

While we found overall effects of site and season on the SIRI, we detected stronger heterogeneity among species in how the total WBC count and NLR responded to these stressors. For example, *A. jamaicensis* had the largest seasonal increase in the NLR compared with other species, with some species such as *St. parvidens* showing a weak decrease in the dry season. Frugivorous species also differed in habitat effects on total WBC counts. Similarly, both *D. rotundus* and *A. jamaicensis* had elevated SIRI in the smaller fragment, despite having highly contrasting diets. Such examples illustrate how the haematological response of species to unfavourable conditions do not map clearly onto dietary guilds, even though foraging ecology plays an important role in diverse aspects of bat biology, including but not limited to demographic responses to land conversion and immunological variation [50–56,61]. This finding underscores caveats about traditional guild assignments, as species may not fit neatly into such categories [46,47], and species-level differences in the immunological response to environmental conditions may be driven by other foraging factors, such as degree of dietary specialization [100].

Future work is needed to better understand the characteristics that predispose species to have different immunological outcomes in response to land conversion and seasonality [25,101]. Reproductive phenology may be one such candidate trait, given some concordance between the seasonal distribution of reproductive activity in our data and species-specific effects of the dry season on cellular immunity. For example, in *A. jamaicensis*, for which we found elevated NLRs in the dry season, we only observed pregnant females in this same season, and lactation was more common in this period than in the wet season (electronic supplementary material, figure S4); this reproductive phenology is similar to that found elsewhere in the species range [102]. Roosting ecology could be another relevant trait-based predictor, as less-ephemeral roosting sites (e.g. caves versus vegetation) could decrease vulnerability to environmental stressors [54,103]. Body mass could also play a role in distinguishing responses of immunity to stressors, given the potential for allometric scaling in immune defence [104,105]; some of our species with stronger responses to seasonality and habitat were relatively large. Ultimately, more comprehensive sampling across host species will be needed to facilitate a trait-based understanding of immune responses to such stressors.

Although our work identifies community-wide and species-specific immunological responses to land conversion and seasonality, we note several limitations. We here relied on blood smears to quantify bat immunity, although total and differential WBC counts are simple measures of the cellular immune system and, particularly with manual slide reading, are subject to substantial variability [73,106]. Future work is needed to understand how more specific components and diverse arms of the immune system respond to land conversion and seasonality, although such efforts will still depend on tools suited to small blood samples and multiple non-model organisms. Approaches such as proteomics may be especially useful here, given its ability to use very small plasma volumes and without species-specific reagents [64,107], albeit the limited availability of appropriate genome annotations can still pose barriers to use across phylogenetically diverse host communities [108]. The variability inherent in haematological data may also explain the relatively small effect sizes observed alongside lack of support for interactive effects of land conversion and seasonality on immunity. Sample size may also play a role here, as we were unable to evenly sample each species in both sites during both seasons due to logistical constraints and factors such as roost abandonment (electronic supplementary material, figure S3). Similarly, whereas we focused on a single year of field data, effects of habitat fragmentation on host immunity and infection can take years to manifest [109], and multi-year sampling will be necessary to differentiate consistent seasonal effects from annual idiosyncrasies [23]. Future work with more even and long-term host sampling, using more holistic measures of immunity, will play a key role in robustly testing if outcomes such as inflammation are elevated in fragmented habitats during unfavourable periods or reproductive seasons [8,23,24]. Our findings here thus provide initial support for the independent effects of these factors that are preconditions for more interactive relationships, and our results more broadly demonstrate the importance of partitioning both within- and across-species effects when considering how environmental change affects wildlife immunity.

## Data Availability

Individual-level data and R code to reproduce primary analyses are available from the Dryad Digital Repository [110]. Supplementary material is available online [111].
